# The gut microbiome: a key player in the complexity of amyotrophic lateral sclerosis (ALS)

**DOI:** 10.1186/s12916-020-01885-3

**Published:** 2021-01-20

**Authors:** Sarah L. Boddy, Ilaria Giovannelli, Matilde Sassani, Johnathan Cooper-Knock, Michael P. Snyder, Eran Segal, Eran Elinav, Lynne A. Barker, Pamela J. Shaw, Christopher J. McDermott

**Affiliations:** 1 Sheffield Institute for Translational Neuroscience, University of Sheffield, Sheffield, UK; 2 Stanford Center for Genomics and Personalized Medicine, Stanford University School of Medicine, Stanford, USA; 3 Department of Computer Science and Applied Mathematics, Weizmann Institute of Science, Rehovot, Israel; 4 Department of Immunology, Weizmann Institute of Science, Rehovot, Israel; 5 Division of Cancer-Microbiome Research, DKFZ, Heidelberg, Germany; 6 Centre for Behavioural Science and Applied Psychology, Sheffield Hallam University, Sheffield, UK

**Keywords:** Amyotrophic lateral sclerosis, ALS, Microbiome, Disease modifiers, Microbial metabolites, Microbial

## Abstract

**Background:**

Much progress has been made in mapping genetic abnormalities linked to amyotrophic lateral sclerosis (ALS), but the majority of cases still present with no known underlying cause. Furthermore, even in families with a shared genetic abnormality there is significant phenotypic variability, suggesting that non-genetic elements may modify pathogenesis. Identification of such disease-modifiers is important as they might represent new therapeutic targets. A growing body of research has begun to shed light on the role played by the gut microbiome in health and disease with a number of studies linking abnormalities to ALS.

**Main body:**

The microbiome refers to the genes belonging to the myriad different microorganisms that live within and upon us, collectively known as the microbiota. Most of these microbes are found in the intestines, where they play important roles in digestion and the generation of key metabolites including neurotransmitters. The gut microbiota is an important aspect of the environment in which our bodies operate and inter-individual differences may be key to explaining the different disease outcomes seen in ALS. Work has begun to investigate animal models of the disease, and the gut microbiomes of people living with ALS, revealing changes in the microbial communities of these groups. The current body of knowledge will be summarised in this review. Advances in microbiome sequencing methods will be highlighted, as their improved resolution now enables researchers to further explore differences at a functional level. Proposed mechanisms connecting the gut microbiome to neurodegeneration will also be considered, including direct effects via metabolites released into the host circulation and indirect effects on bioavailability of nutrients and even medications.

**Conclusion:**

Profiling of the gut microbiome has the potential to add an environmental component to rapidly advancing studies of ALS genetics and move research a step further towards personalised medicine for this disease. Moreover, should compelling evidence of upstream neurotoxicity or neuroprotection initiated by gut microbiota emerge, modification of the microbiome will represent a potential new avenue for disease modifying therapies. For an intractable condition with few current therapeutic options, further research into the ALS microbiome is of crucial importance.

## Background

Amyotrophic lateral sclerosis (ALS) is a fatal neurodegenerative condition characterised by progressive loss of motor neurons. No effective neuroprotective therapy exists; median survival is 2 to 3 years from symptom onset, but there is considerable variation in individual outcomes [1]. Current understanding does not explain the observed heterogeneity in those affected.

In recent years, much progress has been made in elucidating genetic mutations (e.g. within *C9ORF72* and *SOD1*) associated with ALS [2–6]. In certain cases, particular phenotypes are associated with specific genetic variants, including young age of onset [7] and cognitive impairment [8]. However, significant heterogeneity, even within families of patients suffering monogenic disease, suggests that environmental risk factors play a role. Indeed, both sporadic and monogenic ALS are thought to result from a multi-step process based on age-related incidence [9].

Some evidence for a role of specific environmental risk factors (e.g. military service) has been found [10–16]. However, most of those exposed do not develop ALS, and therefore, individual environmental factors likely contribute little to overall disease risk. Interestingly, all proposed environmental factors could potentially impact the gut microbiota and its collective functions. Therefore, the gut microbiome could represent an integrator of the overall environmental contribution to neurodegeneration development.

The most recent studies in *SOD1* and *C9ORF72* model mice have indicated a disease-modifying role for the gut microbiome, with disease severity correlated to particular gut microbe communities [17, 18]. In addition to modifying the risk of developing ALS, it is also possible that a patient’s microbiota can offer protective or deleterious effects, slowing or hastening disease progression, e.g. by impacting systemic inflammation.

The human microbiome refers to the collective genomes of all microbes (i.e. bacteria, archaea, viruses and fungi) that live upon or within the human body, with the community of microbes themselves known as the microbiota. A person’s microbiome contains ~ 150-fold more genes than their human genome [19], so probing the microbiome in a meaningful way poses technical challenges. Nonetheless, recent advances in DNA sequencing technologies have allowed scientists to explore roles of resident microbial communities in health and disease.

Since most of the human microbiome resides within the intestine, several studies in common neurological conditions have focussed on the microbiome of the gastrointestinal tract. Intestinal microbial composition can impact development and progression of disease. For example, gut microbes produce biologically active metabolites and may also modify the absorption of nutrients and drug bioavailability. Particular focus has been given to a proposed bidirectional gut-brain-axis in which the gut microbiome and the CNS engage in biochemical signalling, and the role that the microbiome may play in neurodegenerative diseases such as Alzheimer’s and Parkinson’s diseases [20]. Most recently, investigations have begun to seek links between the gut microbiome and ALS. Small cohorts and methodology limit many of these studies. Nonetheless, differences have been identified between the gut microbiome of ALS patients and controls, supporting further research in this area.

The aim of this article is to review current literature on the microbiome and ALS, and to outline putative mechanisms by which the gut microbiome may impact on the condition. The primary research studies reviewed were selected using the following search terms: “amyotrophic lateral sclerosis” and “microbiome”, “amyotrophic lateral sclerosis” and “microbial”, “motor neuron disease” and “microbiome” in PubMed, up to 26 July 2020 (no lower date limit).

## Main text

### The gut microbiome is linked to ALS, current evidence: mouse models

The first study of an ALS mouse model identified disease-specific damage to intestinal tight junctions, increased gut permeability and reduced levels of the butyrate-producing bacteria *Butyrivibrio fibrisolvens*, in the SOD1^G93A^ mouse [21]. Butyrate is implicated in modulating the immune response, as illustrated in the neuroinflammation section below. In 2017, the same group performed an interventional study attempting to alleviate symptoms through treatment with sodium butyrate [22]: SOD1^G93A^ mice receiving butyrate supplementation developed improved intestinal barrier function and showed delayed weight loss and death compared to untreated controls.

A recent longitudinal study of the SOD1^G93A^ model identified dysbiosis in the pre-symptomatic stage with increased within-sample variance in faecal pellets, but not in ileocolic content [23]. In contrast, an investigation into the impact of short-term vagus nerve stimulation (VNS) on the microbiome of a milder SOD1 mutant model revealed no difference between disease and WT mice at the timepoint investigated, 1 month before symptom onset [24]. No effect on the gut microbiome was seen post VNS.

A 2019 study of the gut microbiome in the SOD1^G93A^ mouse model and in ALS patients represents a major step forward for the field. This is the first study to probe the functional signature of gut microbiome in ALS using shotgun metagenomics. This study identified changes in gut microbiome correlating with biological activity of a microbial metabolite, nicotinamide, possibly modulating the expression of mitochondrial genes in the spinal cord. Notably, detected changes correlated with disease severity in both transgenic mice and human patients [17].

Recently, a study of *C9ORF72*-null mice showed that loss of *C9ORF72* resulted in a pro-inflammatory phenotype which was ameliorated when the gut microbial burden was reduced [18]. The GGGGCC-repeat expansion of *C9ORF72* is the most common genetic cause of ALS [5, 6], and although this genetic change is not thought to be a pure loss-of-function mutation, reduced expression of endogenous *C9ORF72* is also a feature of the human disease [25]. Nonetheless, the murine model used in this study is much less well characterised than the SOD1^G93A^ transgenic mouse model and questions have been raised on its relevance to human ALS.

Studies investigating the microbiome in mouse models of ALS are summarised in Table 1. There are key limitations to these studies, often involving small numbers of animals and 16S rRNA gene sequencing (discussed later). Caution should be drawn before extrapolating findings in mice to the human ALS due to specific limitations of the models [26] and more generally the poor translation rates of findings in rodents to humans to date [27, 28]. Regarding microbial communities, the highly controlled environments of animal facilities poorly replicate those people with ALS reside in, diverging models still further from the human situation.

**Table 1:** Studies investigating the microbiomes of mouse models of ALS

Study	Animal model	Numbers and phenotypes	Intervention/Hypothesis generating	Sample	Methodology	Outcomes
Wu, S., et al. 2015 [21]	SOD1^G93A^	3 mutant, 3 WT in each analysis.	Hypothesis generating	Faecal	16S microbe identification method on extracted DNA	Structural changes in intestines of ALS model mice: Abnormal tight junction structure; reduced levels of ZO-1 and E-cadherin proteins. Increased gut permeability. Increased number of abnormal Paneth cells. Decreased level of defensin 5 alpha (antimicrobial peptide). Changes in ALS mouse model microbiota. Reduction in butyrate-producing bacteria: *Escherichia coli*, *Butyrivibrio fibrisolvens* and *Fermicus*.
Zhang, Y.G., et al. 2017 [22]	SOD1^G93A^	~ 9 per group in each analysis NB: in most cases groups are SOD1^G93A^ vs SOD1^G93A^ plus butyrate, with WT controls included in some analyses.	Interventional	Faecal	16S microbe identification method on extracted DNA. Intervention: 2 groups, one with 2% sodium butyrate added to water stock, the other without.	Butyrate treatment: Delayed disease onset and increased lifespan. Restored levels of butyrate-producing bacteria in gut. Reduced intestinal permeability and corrected structural changes in the ALS model.
Haney, M.M., et al. 2018 [24]	SOD1^G93A^ (mild form with low expression of transgene)	30 mutant, 30 WT. All 5 month-old animals, which is 1 month prior to disease onset in this model.	Hypothesis generating	Faecal	16S microbe identification method on extracted DNA. Ventral neck surgery enabling stimulation of vagus nerve for 1 h. Control treatment groups: surgery plus 1 h sham treatment and no surgical intervention. Faecal pellets collected prior to surgery (day 0) and 8 days later.	Found no difference between the gut microbiomes of the mutant and WT animals at any time point. Found no difference between the gut microbiomes of any treatment group. Therefore conclude that short vagus nerve stimulation has no long-term impact on composition of gut microbiota.
Blacher, E., et al. 2019 [17]	SOD1^G93A^	n numbers vary from 5 to 62 depending on analysis.	Both	Faecal	16S microbe identification method and shotgun metagenomic sequencing on extracted DNA. Intervention: treatment with *Akkermansia muciniphila*.	Identified changes in the gut microbiota of ALS model mice prior to onset of motor dysfunction. Correlate 11 bacteria with disease severity. Evidence that *Akkermansia muciniphila* has a protective effect on the host whereas *Ruminococcus torques* and *Parabacteroides distasonis* are associated with increased disease severity. *Akkermansia muciniphila* therapy improves disease outcomes. Observed differences in microbiota in different facilities.
Figueroa-Romero, C., et al. 2019 [23]	SOD1^G93A^	~ 8 per group for each separate comparison	Hypothesis generating	Faecal	Longitudinal study with the following aspects assessed in parallel: gut microbiome, immunophenotyping, motor function testing and histology of tissue samples. 16S microbe identification method on extracted DNA	Identified changes in the gut microbiota of ALS model mice prior to onset of motor dysfunction, muscle atrophy and activation/expansion of immune cells. Found evidence that microbiome changes and immune responses are related. Noted that SOD1^G93A^ mutant mice have different life expectancies at different facilities.
Burberry et al. 2020 [18]	*C9ORF72*-null mutant mice (+/+, +/−, −/−)	Range between experiments from 10 to 114 of a given mutant genotype, but mostly between 10 and 25.	Both	Faecal	The following aspects were analysed: Gut microbiome, immunophenotyping, inflammatory markers, motor function. 16S microbe identification method on extracted DNA. Faecal transfer intervention post antibiotic treatment.	Evidence that gut microbiota plays role in pathology of *C9ORF72* genetic subtype of ALS: Inflammation/immunological phenotypes significantly reduced when certain bacterial groups are absent or at low abundance. Noted that the heterozygote and double negative *C9ORF72*-null mutant mice have different life expectancies at different facilities.

### The gut microbiome is linked to ALS, current evidence: human studies

Human studies of the gut microbiome in ALS have yielded equivocal findings. Two identified a reduced Firmicutes/Bacteroidetes ratio in patients [29, 30], whilst a third reported the opposite [31]. Nonetheless, those studies detected evidence of dysbiosis in ALS, although they utilised small cohorts (6, 5 and 8 ALS patients, respectively) and one [30] was confined to a specific subgroup of patients with gastrointestinal symptoms. In contrast, a larger 2018 study comparing 25 patients with 32 controls discovered no substantial changes in the gut microbiome including no changes in the Firmicutes/Bacteroidetes ratio [32], though slightly higher diversity was found in ALS samples.

The largest study to date compared 50 patients and 50 age- and sex-matched controls. The abundance of various microbial genera was significantly altered in ALS compared to controls [33] and disease progression coincided with reduced microbial diversity, possibly secondary to dietary changes caused by increased disability. This highlights the importance of monitoring diet in study design. The group also trialled a 6-month probiotic intervention and found no observable benefits.

This year, one study comparing 49 probable/definite ALS cases and 50 controls found no change in the Firmicutes/Bacteroidetes ratio in ALS patients compared to controls [34]. In contrast, the most recent study found a reduced Firmicutes/Bacteroidetes ratio and increased species diversity associated with ALS samples compared to healthy controls (*n* = 20, both groups) [35]. Despite finding no differences between the disease and control groups, the larger study did report that within the ALS group, a higher Firmicutes/Bacteroidetes ratio was associated with increased risk of death, as was higher species diversity [34].

Antibiotics significantly modify the balance of gut microbial species [36]; hence, correlations between antibiotic usage and seemingly unrelated diseases are of interest. A 2019 study demonstrated that the use of antibiotics, especially repeatedly, was associated with increased risk of developing ALS [37]. The study was based on whole-population data for Sweden (2006–2013 period), but limited by the retrospective design and the lack of associated microbiome data to probe for evidence of dysbiosis. Similarly, experiments using the SOD1^G93A^ mouse model showed that repeated exposure to antibiotics was associated with development of a more severe motor phenotype and increased neuronal loss [17]; this effect was specific to the diseased mice and no motor effect was observed in WT mice.

All published human ALS microbiome studies are summarised in Table 2, including two studies investigating microbes residing within the CNS [38, 39]. As those do not explore the gut microbiota, they are not discussed further in the text.

**Table 2:** Studies investigating the ALS microbiome. Includes non-gut microbiome studies not described further in the main text

Study	Numbers and phenotypes	Intervention/ Hypothesis generating	Sample	Methodology	Outcomes
Alonso, R. et al. 2015 [39]	5 ALS and 3 controls (CSF sample analysis), 6 ALS and 4 controls (brain samples)	Hypothesis generating	CSF, brain tissue	PCR, immunohistochemistry and proteomics	Evidence of fungal infection both in CSF and brain tissue of ALS patients. Evidence of some fungal presence also in controls.
Fang, X., et al. 2016 [29]	6 ALS patients, 5 healthy controls	Hypothesis generating	Faecal	16S microbe identification method on extracted DNA	In ALS group (compared to control): Decreased Firmicutes/Bacteroidetes ratio Significantly increased *Dorea* genus Significantly reduced *Oscillibacter*, *Anaerostipes*, Lachnospiraceae
Rowin, J., et al. 2017 [30]	5 patients, 4 definite ALS, one Brachial Amyotrophic Diplegia vs control reference ranges (*n* = 96)	Hypothesis generating	Faecal	16S microbe identification method on extracted DNA	Decreased diversity in disease group compared to healthy reference. Low Firmicutes/Bacteroidetes ratio in 3 of 4 ALS patients.
Brenner, D., et al. 2018 [32]	25 ALS patients with 32 age- and gender-matched healthy controls	Hypothesis generating	Faecal	16S microbe identification method on extracted DNA, predicted metagenomes	Only 1 genus found statistically different, Ruminococcaceae, out of 336 considered and non-significant higher diversity in ALS group.
Zhai, C.D., et al. 2019 [31]	8 ALS patients, 8 healthy controls	Hypothesis generating	Faecal	16S microbe identification method on extracted DNA, evaluation of metabolite concentrations by spectrophotometry (SCFAs, NO2-N/NO3-N, GABA)	Report the following trends but no mention of significance: Firmicutes/Bacteroidetes ratio increased in ALS group. *Methanobrevibacter* increased, *Faecalibacterium* and *Bacteroides* decreased in ALS patients. Small differences in average metabolite concentrations of ALS and control groups reported.
Sun, J., et al. 2019 [37]	2484 ALS and 12,420 age-matched and sex-matched controls.	Hypothesis generating	Medical records	Nested case–control study using several Swedish national registers. Conditional logistic regression model.	Antibiotic use found to be associated with higher risk of ALS. Findings irrespective of reason for antibiotic administration (respiratory, urinary tract and soft tissue infections)
Alonso, R. et al. 2019 [38]	11 ALS tissue samples from brain and spinal cord, control data appears to be from a previous study (data from 9 individuals used in a PCA).	Hypothesis generating	Brain and spinal cord tissues	PCR, next generation sequencing and immunohistochemistry	All methods employed showed evidence of bacteria present in brain and spinal cord tissue of ALS patients.
Blacher, E., et al. 2019 [17]	37 ALS patients, 29 healthy controls.	Hypothesis generating	Faecal	Shotgun metagenomic sequencing. Metabolomics of blood serum.	Microbiome composition of ALS group significantly different to control group. Groups significantly different in global bacterial gene content with ALS group deficient in several genes associated with metabolism of tryptophan and nicotinamide. Various metabolites differentially expressed in ALS sera compared to controls, including metabolites of the tryptophan-nicotinamide pathway. Proposed link between bacteria-derived nicotinamide and protection from ALS.
Di Gioia, D., et al. 2020 [33]	50 ALS, 50 healthy age/sex-matched controls	Both	Faecal	16S microbe identification method on extracted DNA. Quantitative PCR for selected microbes. Preliminary comparison of disease vs control samples using PCR-DGGE. Treatment (6 month) with *Lactobacillus fermentum*, *Lactobacillus delbrueckii*, *Lactobacillus plantarum*, *Lactobacillus salivarius*.	Levels of various microbial genera found to be significantly increased/decreased in abundance in ALS samples compared to healthy controls. Significant decrease in OTU number in probiotic trial longitudinal samples i.e. richness of gut microbiota declines as disease progresses. Probiotic treatment did alter gut microbiota, but without moving biodiversity towards that seen in controls. No impact on disease progression observed in probiotic-treated group.
Ngo, S.T., et al. 2020 [34]	49 probable/ definite ALS, 51 healthy controls	Hypothesis generating	Faecal	16S microbe identification method on extracted DNA. Taxa comparison. Predictive metagenomic analyses.	Overall no difference between faecal microbiomes of ALS patients and healthy controls. However, a subset (four) of ALS samples showed differences in beta-diversity. Higher species diversity associated with faster disease progression. Increased F:B ratio also linked to accelerated decline. Considered other clinical, metabolic and anthropometric features and none correlated with microbiome.
Zeng, Q., et al. 2020 [35]	20 probable/definite ALS, 20 healthy controls	Hypothesis generating	Faecal	16S microbe identification method on extracted DNA. Shotgun metagenomic sequencing on 10 ALS and 10 control samples. Metabolomic analysis (all 40 samples).	Reduced F:B ratio in ALS samples compared to controls. Evidence of higher OTU diversity and some change in structure of intestinal microbiota community in ALS samples. Decreased function of various metabolic pathways inferred from sequencing data, backed up to a degree by the metabolic data.

Whilst data are accumulating, many results are discordant. Studies are largely exploratory and cohort numbers have been relatively small which, in view of significant interindividual variability, may preclude identification of relevant microbiome features. These findings, alongside those reported in animal studies, demonstrate the importance of considering within-group differences across the ALS population as well as between-group changes between ALS and controls. It is possible that specific microbiome signatures may be either protective or toxic in different individuals. Future studies will need to significantly increase sample size and combine the microbiome profile with detailed measurement of interacting partners such as the host genome, nutrition and medication.

### A note on methodologies

We have reviewed all published studies on the gut microbiome in ALS. Most utilised 16S rRNA gene sequencing. However, recent technological developments have made more in-depth microbiome sequencing feasible and affordable. Shotgun metagenomic sequencing is becoming the standard due to its increased resolution, enabling not only identification of microbes at species levels, but also discovery of novel microbes. Furthermore, this technology facilitates functional analyses and critically is able to account for horizontal gene-transfer events, which inferred functional analyses from 16S-derived data cannot do [40–44]. When it is considered that microbes often share functional enzymes, then a functional measure is clearly more informative than identifying specific species. We do accept that sometimes, due to cost or sample quality, 16S rRNA sequencing may be the only option and it is important to note that efforts have been made to predict function from 16S data with some success [45]. The advantages offered by shotgun metagenomic sequencing are reviewed elsewhere [44, 46], but it is appropriate to highlight the different analytical techniques used in studies (Tables 1 and 2). Thus far, only Blacher et al. [17] have reported data based on metagenomic sequencing.

Beyond metagenomics, other methods are being developed to measure function of gut microbes including metabolomics, metatranscriptomics and metaproteomics [47]. A multi-omic approach to the microbiome is likely to improve power for detection of biology just as a multi-omic approach to the host is becoming increasingly important; this is illustrated by the use of metabolomics in the study described above [17]. Significant innovation is also occurring in analysis methods. Microbes can interact functionally even without gene-transfer for example, if a collection of microbes each encodes an enzyme which produces a substrate for another enzyme carried by a different microbe. It is possible to predict the presence and effect of the so-called biosynthetic gene clusters using ClusterFinder [48].

### The gut microbiome may contribute to ALS heterogeneity: putative roles

**Fig. 1:**
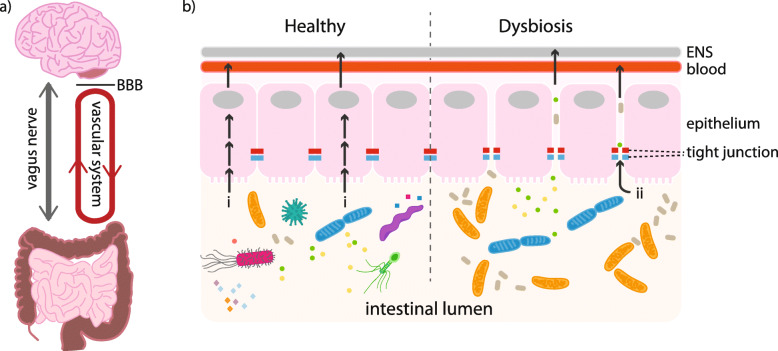
Pathways linking gut microbial function to changes in the CNS. **a** Macro-scale pathways: The enteric nervous system (ENS) intrinsic to the gut is connected to the central nervous system directly via the vagus nerve. As such, any microbe-derived metabolite that accesses the ENS has the potential to travel to and impact the brain and spinal cord. Likewise, an extensive network of blood capillaries collects nutrients absorbed from the gut for transfer around the body. Microbial metabolites that access the bloodstream can impact any part of the body, though still need to breach the blood-brain-barrier (BBB) to access the CNS. **b** Transit across the intestinal epithelium: In a healthy gut with functional tight junctions, selective uptake of contents of the intestinal lumen occurs across the epithelial cells (route “i”). Dysbiosis of the gut microbiota can damage the structural integrity of the epithelial barrier allowing uncontrolled transit of metabolites and other luminal contents to pass into the body (route “ii”)

Various ways in which the gut microbiota and CNS may interact have been proposed, with communication routes referred to as the gut-brain-axis (Fig. 1). It has been suggested that microbes might influence the CNS directly, through production of neuroactive metabolites released into the systemic circulation or via the enteric nervous system [49, 50]. Possible indirect connections include modulation of CNS inflammation [51, 52], alteration of nutrient absorption [53, 54] and modification of the metabolism of exogenous drugs [55]. Evidence supporting two of these putative mechanisms has emerged from recent literature: Blacher et al. [17] provided data supporting the role of microbial neuroactive metabolites in ALS, whereas others have found links between gut microbiota, the immune system and ALS [18, 21–23]. Notably, although these mechanisms are not mutually exclusive, no consensus has yet been reached and, in addition to limitations highlighted above, studies are not directly comparable primarily because of differences in analysis methodologies. The last section will detail these two mechanisms and suggest other promising avenues yet to be explored in the field.

### Metabolite modulation of neuronal function

**Fig. 2:**
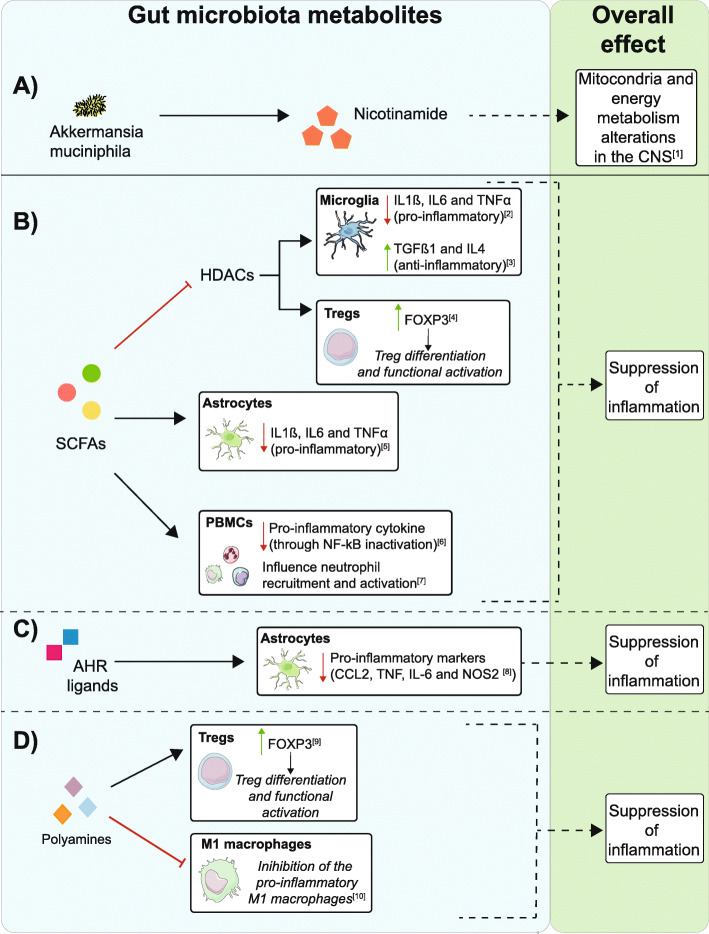
Metabolites produced by microbes found within the gut can influence neuronal health either directly or indirectly via CNS inflammation. **a** Metabolites released by the gut microbiome can enter the system circulation where they can access the CNS; in the case of nicotinamide released by *Akkermansia muciniphila*, this potentially modifies energy homeostasis and oxidative stress [17]. **b**–**d** A number of proposed mechanisms exist by which metabolites produced by microbes found within the gut can influence the immune response and have an effect of the CNS inflammatory state. **b** Short-chain fatty acids (SCFAs) can reduce inflammation by inhibiting HDACs within microglial cells, leading to the downregulation of pro-inflammatory (IL1ß, IL6 and TNFα) and upregulation of anti-inflammatory markers (TGFβ and IL4) [56, 57]. SCFA-mediated HDAC inhibition can also impact Tregs increasing their activity via upregulation of FOXP3 [58, 59]. SCFAs also influence astrocytes, reducing their inflammatory impact through downregulation of IL1ß, IL6 and TNFα [60]. Lastly, SCFAs exert anti-inflammatory effects on different peripheral blood mononuclear cells: they inhibit NF-kB leading to reduced pro-inflammatory cytokine production and immune cell recruitment and activation [61–63]. **c** Aryl hydrocarbon receptor (AHR) ligands can modulate astrocyte activities and give rise to anti-inflammatory properties [64]. **d** Polyamines induce FOXP3 expression in Treg cells promoting their differentiation and activation [65]. These molecules can also inhibit inflammatory macrophages (M1) thereby preventing macrophage-induced inflammation [66]

The strongest evidence for microbe-derived neuroactive metabolites modifying ALS comes from the study of Blacher et al. [17] which focused on the protective effect of nicotinamide released by *Akkermansia muciniphila* (Fig. 2a). The authors argued for the role of bacterial metabolites in modulating neurodegeneration; they were able to manipulate disease severity in SOD1^G93A^ mice via supplementation with gut microbial species. *Ruminococcus torques* and *Parabacteroides distasonis* were associated with increased severity, whereas *Akkermansia muciniphila* improved outcomes. Mechanistic links were explored through untargeted serum metabolomic-profiling, which identified changes in biological pathways featuring nicotinamide in mice receiving *Akkermansia muciniphila* supplementation. Importantly, direct nicotinamide administration replicated the beneficial effects of *Akkermansia muciniphila*. Bulk RNA-sequencing of spinal cord tissue from SOD1^G93A^ mice treated with nicotinamide or *Akkermansia muciniphila* revealed changes in mitochondrial function and oxidative stress pathways. These observations were corroborated in ALS patients: bacterial genes associated with tryptophan and nicotinamide synthesis were decreased in patients’ stool samples, and targeted metabolomic profiling revealed a lower nicotinamide concentration in serum and CSF compared to controls.

The study authors argued for a pathway from gut microbiome function to motor neuron death via metabolites secreted into the host circulation. Nicotinamide is the precursor of NAD and NADP, coenzymes necessary for appropriate functioning of energy transducing and antioxidant pathways as well as other cellular signalling mechanisms [67], many of which have been implicated in ALS-related neurodegeneration [15, 68].

### Modulation of CNS inflammation

Neuro-inflammation is a well-characterised ALS pathological mechanism, defined as a complex dysregulation amongst both resident and peripheral immunological cells [15]. Its main features are activation of microglia and astrocytes, infiltration of T cells and upregulation of pro-inflammatory cytokines. The human microbiome exerts a considerable influence on the immune system: in development when immune cells begin to discriminate between necessary commensals and harmful pathogens, but also in adulthood, as microbes contribute to immune homeostasis [69, 70]. In fact, germ-free (GF) mice display a broad range of immunological abnormalities [69, 71]. To date, the underlying mechanisms responsible for crosstalk between microbiome and the immune system are not fully understood. Several bacterial-derived molecules are thought to be immunological modulators (Fig. 2b-d) including short chain fatty acid (SCFAs), aryl hydrocarbon receptor (AHR) ligands, polyamines and polysaccharides [72]. SCFAs such as butyric acid, propionic acid and acetic acid are products of dietary fibre metabolism by the gut microbiome, mainly from Bacteroides and Firmicutes [73]. SCFAs are known to mediate regulatory T cell (Treg) induction through histone deacetylase inhibition (see Fig. 2). The inverse is also seen in mice where depletion of the Treg subset is associated with a significant increase in gut Firmicutes [74]. Although few studies have investigated changes in ALS microbiota compared to healthy individuals, disequilibrium of the Firmicutes/Bacteroides ratio has been reported [29–31, 33]. As these bacteria are the main producers of SCFAs, it could be speculated that alterations of these metabolites may affect ALS patients by directly acting on CNS cells and/or indirectly through immune system modulation. One of the SOD1^G93A^ studies is consistent with this, where butyrate supplementation appeared to alleviate the clinical features of ALS [22]. The same group reported earlier that, prior to symptom onset, this mouse model is characterised by a leaky intestine, increased intestinal permeability and reduced abundance of butyrate-producing bacteria [21]. This is associated with increased levels of inflammatory IL-17 and abnormalities in Paneth cells, which are crucial for host-microbiome interaction and immunity [21]. Butyrate supplementation in SOD1^G93A^ mice significantly decreased intestinal permeability, reduced the number of abnormal Paneth cells and increased life span [22]. Moreover, a longitudinal study showed evidence of dysbiosis (in particular alterations in Bacteroides and Firmicutes) in SOD1^G93A^ mice beginning before disease onset (37 days) and persisting until end-stage (~ 150 days). Concomitantly, alterations in the immune system were reported. These were limited to the peripheral system in early disease stages but affected the CNS later during disease course. They also documented both positive and negative correlations between microbiome dysregulation and spinal cord inflammation [23].

Microbiota-deficient mice also provide evidence linking the gut microbiome to the CNS through the immune system. Germ-free (GF) and antibiotic-treated mouse models develop immunological abnormalities and neurodegeneration [75]. In this context, microglia functions appear to be strictly connected to the gut microbiome [51] as GF or antibiotic-treated mice exhibit aberrant microglia maturation and functional impairments [76], characterised by immature phenotype, morphology alterations, diminished responsiveness to lipopolysaccharides and overall attenuated immune activation. A C9ORF72-null mouse study suggests that dysregulated microflora, characterised by immune-stimulating bacteria, reduce mice survival by inducing detrimental peripheral inflammation and microglia activation. However, antibiotic treatment or the transplantation of protective microbiota improved symptoms [18]. Astrocyte activity is also regulated by the gut microbiome through an aryl hydrocarbon receptor (AHR)-mediated mechanism involving type I interferon signalling [64]. This leads to a different hypothesis: that proinflammatory signals driven by gut microbiota may be key for physiological functioning of glia, which in turn maintain neuronal health. Abnormal function of microglia [77] and astrocytes [78] has been linked to motor neuron death. Confirmation of this hypothesis will depend on identification of direct correlations between gut microbiome features, disease severity and CNS inflammation in a single model system; it should be noted that an unbiased metabolomic study did not identify a significant inflammatory signal [17].

Given the potential link between the microbiome, the immune system and ALS, a clinical trial is currently ongoing to evaluate the effects of faecal microbial transplantation in 42 patients (NCT03766321). The investigators anticipate that microbiome modulation will increase the proportion and suppressive abilities of immune-suppressive Treg cells leading to the establishment of a neuroprotective anti-inflammatory environment [79]. Moreover, several experimental medicine trials are ongoing with the aim of modulating the immune response using small molecules (e.g. MIROCALS (NCT03039673)) [80]. Should analogous results be achieved by altering the composition of the gut microbiome, this would be an attractive alternative approach.

### Impact on nutrition

Much research has focused on the role of the gut microbiome in human nutrition [81]. Microbes resident in the gut can alter the quantity of nutrients extracted from food and even synthesise key nutrients themselves, including vitamin K and various B vitamins [82]. Experiments transplanting the gut microbiota of obese mice or humans into germ-free animals of a healthy weight produced weight-gain demonstrating that metabolic phenotypes can be transmitted via the gut microbiome [83, 84]. Weight maintenance has been highlighted as critical to the clinical outcome in ALS, with rapid weight loss associated with faster disease progression [85]. Ongoing work is focused on modifying the diet in patients with a view to slowing disease progression. It is possible that, ultimately, this work will need to investigate both diet and the modulating effect of the gut microbiome on nutrition. Furthermore, Di Gioia et al. showed a decline in microbial diversity in ALS samples over the course of a probiotic study, the only longitudinal microbiome data reported in this disease group to date [33]. This is not surprising as the progression of ALS is associated with declining chewing, swallowing and self-feeding functions, all of which are likely to impact the gut microbiota, in addition to reduced physical activity and likely environmental changes e.g. associated with leaving the workplace and/or becoming increasingly house-bound. Indeed, during the later stages of the disease, many patients opt to be fed enterally through formulated foods, a dramatic dietary change likely to impact the microbial communities of the gut. Therefore, it is also important to consider the impact disease progression has on the gut microbiota. This is critical when considering differences seen between ALS and control samples, with caution necessary when inferring aspects of the microbiota may play a role in disease development.

### Impact on drug efficacy

The gut microbiome can also impact disease via metabolism of enterally delivered drugs. A recent study revealed that the primary treatment for Parkinson’s disease, L-DOPA, is metabolised by gut microbial species which vary in abundance between individuals [55]. Prevalence of a microbial gene encoding an enzyme proficient in decarboxylating L-DOPA correlated positively with the drug dosage required to provide symptomatic relief and with L-DOPA concentration in the systemic circulation. In 2019, a study assessed the capacity of a panel of gut bacteria to metabolise a selection of commonly prescribed drugs including Riluzole, the only drug that has shown to confer a survival benefit in ALS [86]. Riluzole was significantly metabolised by 40 of the bacteria screened [87] many of which are known to vary in prevalence in the human population. The plasma concentration of Riluzole reportedly shows low within-patient variability compared to relatively high interpatient variability [88], which is not explained by differences in metabolism after gut absorption [89]. Modification of Riluzole bioavailability by the gut microbiome may explain the observed interpatient variability in plasma levels.

### Impact on non-motor ALS symptoms

Finally, the microbiome has been linked to other symptoms known to impact subgroups of ALS patients, such as depression, anxiety and constipation [90]. The gut microbiota can produce various peptides and neurotransmitters that could directly impact mood [49, 90] whilst the brain affects the gut through a variety of mechanisms including stress responses [91]. Unravelling the role the gut microbiota play in regulating brain function relating to neuropsychiatric conditions has only just begun [90, 92], but there is potential for this to be a means of improving quality of life for ALS patients.

Regarding constipation, another symptom often reported by ALS patients [93], roles for the microbiome in luminal fluid (metabolism of bile acids [94], generation of short chain fatty acids [95, 96] and methane production [97]) as well as mucosal layer of the colon [98] in regulating the absorption of fluids into the bloodstream have all been proposed. Improved management of these symptoms would improve quality of life [99] irrespective of disease progression.

### Integration of microbiome with host genomics

Notably, the gut microbiome is affected by environment, and evidence suggests almost complete independence from host genetics [100]; therefore, the two measures may be usefully combined to describe gene-environment interaction. This could be a powerful tool to approach diseases resulting from a complex interaction of genes and environment. Approaches such as deep learning or other machine learning methodologies may be required to overcome non-linearity. There is potential to provide a personalised medicine approach whereby interventions targeted at the microbiome are tailored to the host’s genome.

## Conclusion

### The missing piece of the puzzle?

It is well established that people with ALS exhibit a wide range of disease severity and whilst some risk factors have been identified, they remain insufficient to fully explain this heterogeneity. The gut microbiome may be crucial in resolving some of these differences due to the variety of ways it could affect disease, both directly and indirectly. Further research is essential to identify relevant microbial players in ALS so that they may be targeted in future therapies seeking to modify gut microbiota to modulate disease progression and improve quality of life. It is likely that such interventions will be personally tailored to patients from different environments and with different genotypes.

## Data Availability

Not applicable (Review).
